# Waist circumference as a marker for screening nonalcoholic fatty liver disease in obese adolescents

**DOI:** 10.1016/j.rppede.2015.10.004

**Published:** 2016

**Authors:** Ana Paula Grotti Clemente, Bárbara Dal Molin, Joana Pereira de Carvalho-Ferreira, Raquel Munhoz da Silveira Campos, Aline de Piano Ganen, Lian Tock, Marco Túlio de Mello, Ana Raimunda Dâmaso

**Affiliations:** a Universidade Federal de Alagoas (Ufal), Maceió, AL, Brazil; b Escola Paulista de Medicina, Universidade Federal de São Paulo (EPM-Unifesp), São Paulo, SP, Brazil

**Keywords:** Aspartate aminotransferase, Alanine aminotransferase, Abdominal fat, Adolescents

## Abstract

**Objective:**

To assess the relationship between the degree of waist circumference (WC) and nonalcoholic fatty liver disease (NAFLD) in obese adolescents of both genders, analyzed according to quartiles of WC.

**Methods:**

Cross-sectional study that involved 247 obese adolescents aged 12–19 years. Mean values of the nutritional parameters and serum analyses were compared with the groups using the independent *t*-test. Pearson correlation coefficient was used to determine the relationship of the parameters studied. Chi-square test for trend was used to determine the relationship between the prevalence of the NAFLD and WC quartile by gender.

**Results:**

NAFLD were presented in 60% of the study participants. Obese adolescents in the 3rd and 4th quartiles of WC presented higher prevalence of NAFLD when compared with that in the 1st quartile in both genders. The NAFLD patients had significantly higher values for body weight, BMI (body mass index), BAZ-score (BMI-for-age z-scores), total fat (% and kg), WC, visceral fat, insulin, insulin resistance index (HOMA-IR), aspartate aminotransferase and alanine aminotransferase, when compared with non-NAFLD obese adolescents.

**Conclusions:**

In conclusion, the results presented here suggest that an increase in WC can reliably predict the risk of NAFLD in obese adolescents. This is a low cost and easy-to-use tool that can help in screening in adolescents.

## Introduction

Nonalcoholic fatty liver disease (NAFLD) is the most common cause of chronic liver disease worldwide and has been recognized as the early manifestation of obesity and metabolic syndrome.^1^ NAFLD is characterized by the accumulation of large droplets of triglycerides within hepatocytes in the absence of chronic alcohol consumption.^2^ Currently, NAFLD affects between 3% and 11% of the pediatric population reaching the rate of 46% among overweight and obese children and adolescents.^3^ Indeed, previous study from our group found that NAFLD affected 52% of obese adolescents.^4^

NAFLD development is influenced by multiple genetic and environmental factors. Currently, NAFLD is recognized as the hepatic component of metabolic syndrome due to its strong association with obesity, dyslipidemia, hypertension and insulin resistance index (HOMA-IR). It has long been known that there is a highly significant relation between NAFLD and insulin resistance. A study developed with Japanese children suggested that hyperinsulinemia was the most important clinical manifestation associated with NAFLD.^5^ Moreover, insulin resistance is accepted as the main pathophysiologic factor in developing NAFLD.^6^

In agreement, de Piano et al.^7^ verified that adolescents with visceral obesity and high HOMA-IR levels presented a higher risk of developing NAFLD, which could lead to the accumulation of lipid in the hepatocytes. In addition, it was demonstrated that each 1-cm increase in visceral adiposity was associated with a two-fold greater risk of NAFLD in obese adolescents.^8^

In fact, the central adiposity is associated with chronic low-grade inflammation, which accelerates insulin resistance and accumulation of hepatocellular fat. Subjects with NAFLD are at risk of developing cardiovascular disease (CVD) through insulin-resistance related mechanisms.^9^ Therefore, it is important to assess visceral adiposity in clinical practices. For assessment of central obesity in young ages, ultrasound and magnetic resonance imaging are available. However, these procedures have some limitations for broad use, such as cost. On the other hand, WC may be a simple clinical and cost-effective tool to be used as a surrogate marker for NAFLD.^10^ WC has been shown to be an inexpensive tool for assessing central obesity in the clinical practice, with excellent correlation with abdominal imaging and high association with CVD risk.^11^ For this reason, WC is one of the diagnostic criteria proposed by the International Diabetes Federation (IDF) in adolescents and has been identified as a valuable predictor of metabolic syndrome and CVD risk.^12^

The relation between NAFLD and atherosclerosis development has been evaluated in pediatric studies.^1^^,^ 9 Fallo et al.^13^ reported that WC was a predictor for NAFLD in their study that included 86 hypertensive obese adults. Another study found that the increased WC and body mass index (BMI) were associated with a significant higher risk of insulin resistance and NAFLD in healthy Koreans adults. In addition, the authors reinforced the importance of using both BMI and WC in clinical practice, because they may be helpful in evaluating the risk of NAFLD and insulin resistance.^14^ Finally, WC measurement has known to predict cardiovascular risk, although its value for NAFLD risk in adolescents has not yet been explored.

Therefore, WC is a convenient measure of abdominal obesity. However, few studies have been performed on the relationship between intra-abdominal fat area and NAFLD risk. Thus, in this study we aimed to assess the relationship between the WC and the presence of NAFLD in Brazilian obese adolescents of both genders, analyzed according to quartiles of WC.

## Method

The study was formally approved by the Committee of Ethics in Research of the Universidade Federal de São Paulo (UNIFESP; protocol no. (#0135/04) and registered as a clinical trial (NCT01358773). Written informed consent was obtained from all potential participants and/or their parents or legal guardians prior to the commencement of the study.

**Figure 1:**
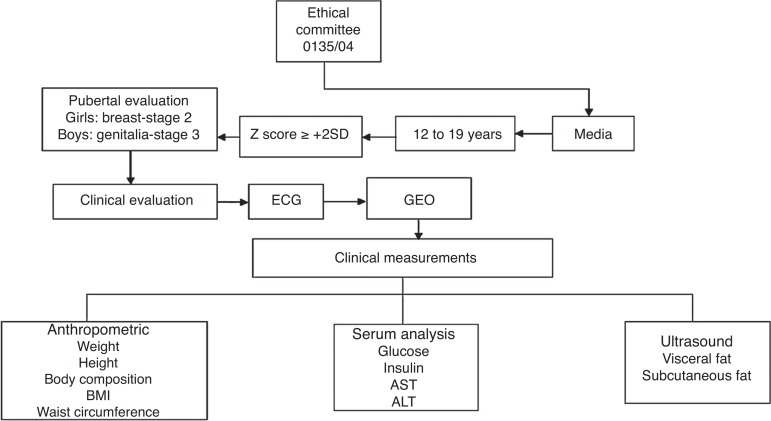
Description of study flow. ECG, electrocardiogram; GEO, Group of Study in Obesity; AST, aspartate aminotransferase; ALT, alanine aminotransferase.

The study flow is shown in Fig. 1. For this cross-sectional study, 247 obese adolescents with aged from 12 to 19 years were included. Data were collected from the screening obese adolescents in the years 2007–2010. Obese adolescents were recruited from Multidisciplinary Obesity Intervention Program outpatient clinic of the Federal University of São Paulo. All patients enrolled in this study were assessed before weight loss therapy. Nutritional status was calculated according to height-for-age Z-score (HAZ) and BMI-for-age values using WHO Anthro-Plus 1.0.4 software. The nutritional diagnosis was based on the BMI-for-age (BAZ) for the children aged more than 5 years and adolescents ≤19 years of age (Z score ≥+2SD), according to the cut-offs defined by World Health Organization.^16^ Non-inclusion criteria were identified as genetic, metabolic or endocrine disease, chronic alcohol consumption (≥20g/day), presence of viral hepatic diseases, previous drug use, and other causes of liver steatosis.

Pubertal stage was established with clinical screening and anthropometric measures were assessed (stature, body mass, BMI and body composition). Ultrasound (US) was performed and blood sample was collected and analyzed for metabolic profile. To exclude influences of diurnal variations, the procedures were scheduled for the same time of the day for all subjects, 8:00AM after an overnight fasting.

All adolescents were examined by a trained physician and pubertal stage was classified according to Tanner scale^15^ for both boys and girls. Girls with breast-stage 2 and boys with genitalia-stage 3 were considered pubertal, whilst those who had yet to attain these stages were classified as non-pubertal.^15^ None of the participants presented early or delayed puberty, although levels of testosterone, luteinizing hormone or follicle-stimulating hormone were not determined.

The body mass was measured (wearing light clothes and without shoes) in a single assessment using a platform scale Filizola™ (Indústrias Filizola S/A, São Paulo-SP, Brazil; model PL 180), with a capacity of 180kg and an accuracy of 100g. The stature was assessed using a stadiometer with a precision of 0.1cm (Sanny, São Bernardo do Campo, SP, Brazil; model ES 2030). BMI values were calculated as the quotient of body mass (kg) and the square of the stature (m). For the determination of WC, subjects were placed in a standing position with the abdomen and arms relaxed alongside the body, and a flexible measuring tape (1mm accuracy) was held horizontally at the midpoint between the bottom edge of the last rib and the iliac crest. The measurements were recorded with the tape applied firmly to the skin but without compression of tissues. Body composition was measured by air displacement plethysmography in a BOD POD body composition system (version 1.69; Life Measurement Instruments, Concord, CA).

Blood samples (10mL) were collected from overnight fasted adolescents by venous puncture and transferred, as appropriate, to heparinized and non-heparinized vials. Plasma glucose was determined with the aid of a commercial kit and a UniCell DXI 800 spectrophotometer (Beckman Coulter, Fullerton, CA, USA), while specific insulin (without C peptide) was determined using an enzyme assay and an Advia 2400/Kovalent analyzer (Siemens, São Paulo, Brazil). Serum levels of hepatic transaminases, alanine aminotransferase (ALT), and aspartate aminotransferase (AST) were analyzed using a commercial kit (CELM, Barueri, Brazil). Insulin resistance was assessed by homeostasis model assessment insulin resistance index (HOMA-IR). HOMA-IR was calculated by the fasting blood glucose (FBG) and the immunoreactive insulin (I): [FBG (mg/dL) × I (mU/L)]/405.

Obese adolescents were divided into quartiles according to WC. For the first quartile, adolescents with WC less than 91.8cm (low WC); for the second quartile, adolescents with WC values between 91.8 and 99cm (moderate WC); for the third quartile, adolescents with WC between 99.1 and 107.5cm (high WC); finally, for the fourth quartile, adolescents with WC above 107.5cm were included.

Statistical analyses were performed using PASW Statistics version 19 (SPSS Inc, Chicago, IL, USA) with the level of significance set at *p*<0.05. Mean values of the nutritional parameters (age, height, weight, HAZ, BMI and WC) and serum analyses (insulin, glucose, ALT and AST) of the non-NAFLD and NAFLD groups, stratified according to gender, were compared using independent *t*-test and the assumptions of homoscedasticity verified using the Levene test. Pearson correlation coefficient was used to determine the relationship between the independent variable nutritional status and biochemical parameters and WC. Chi square test for trend was used to determine the relationship between the prevalence of the NAFLD and WC quartile by gender. Lastly, we performed analysis of covariance, using the presence of NAFLD as factor, and WC as dependent variable. Since other anthropometric variables presented a high degree of correlation with WC, the confounding effects of age and gender were evaluated in the NAFLD group.

## Results

The study enrolled 247 obese adolescents: 90 boys (36.5%) and 157 girls (63.5%). Among the participants 148 (60%) presented NAFLD. The body composition, and anthropometric and biochemical characteristics of the subjects are presented in Table 1.

**Table 1:** Anthropometric, body composition and biochemical parameters of the studied population.

	NAFLD (n=40)	Non-NAFLD (n=50)	NAFLD (n=59)	Non-NAFLD (n=98	NAFLD (n=148)	Non-NAFLD (n=99)	*p* -value Boys	*p* -value Girls	Gender Group a	Gender Group b	Total
Age (years)	16.78±1.63	15.87±1.65	16.94±2.10	16.30±1.80	16.88±1.92	16.15±1.76	0.01	0.04	0.67	0.15	0.02
Weight (kg)	117.07±17.24	100.48±16.08	100.69±15.45	91.13±13.75	107.31±18.02	94.29±15.19	<0.01	<0.01	<0.01	<0.01	<0.01
Height (cm)	174.65±6.77	171.34±8.18	163.37±6.85	162.53±5.93	167.92±8.77	165.50±7.93	0.04	0.41	<0.01	<0.01	0.03
BAZ (Z score)	3.51±0.83	3.04±0.65	3.25±0.80	2.82±0.69	3.36±0.82	2.89±0.68	0.04	0.01	0.07	0.23	<0.01
BMI (kg/m^2^)	38.37±4.95	34.08±4.10	37.71±4.98	34.46±4.52	37.97±4.95	34.33±4.38	<0.01	<0.01	0.52	0.64	<0.01
Total fat (%)	42.92±6.08	38.08±6.04	47.71±4.84	45.20±5.54	45.77±5.84	42.97±6.49	0.01	0.05	<0.01	<0.01	0.01
Fat free mass (%)	57.32±5.98	61.50±6.18	52.28±4.84	54.92±5.43	54.32±5.85	57.16±6.48	0.02	0.03	<0.01	<0.01	0.01
Total fat (kg)	50.72±12.25	38.96±9.96	48.35±10.47	41.37±10.28	49.31±11.22	40.55±10.20	<0.01	<0.01	0.31	0.17	<0.01
Fat free mass (kg)	66.52±9.76	62.58±10.95	52.28±6.97	49.31±5.02	58.04±10.77	53.82±9.83	0.08	0.05	<0.01	<0.01	0.02
WC (cm)	110.30±10.83	99.34±8.17	102.59±10.82	94.71±9.05	105.70±11.42	96.27±9.01	<0.01	<0.01	0.01	0.03	<0.01
Visceral fat (cm)	5.82±1.64	4.30±1.23	4.52±1.31	3.73±1.20	5.05±1.58	3.92±1.24	<0.01	<0.01	<0.01	0.22	<0.01
Subcutaneous fat (cm)	3.60±0.95	3.44±0.88	3.86±1.09	3.69±1.04	3.75±1.04	3.60±1.00	0.41	0.33	0.27	0.07	0.26
Glucose (mg/dL)	92.17±7.99	91.54±6.47	89.91±7.52	89.93±6.58	90.82±7.75	90.47±6.56	0.68	0.98	0.15	0.16	0.70
Insulin (uU/mL)	23.87±12.42	15.98±10.28	20.00±8.33	16.10±10.37	21.56±10.30	16.06±10.30	<0.01	0.01	0.06	0.95	<0.01
HOMA-IR	5.47±2.91	3.64±2.51	4.37±1.90	3.66±2.94	4.81±2.41	3.65±2.79	0.02	0.01	0.04	0.98	0.01
AST, U/L	32.30±14.00	24.36±6.23	22.08±4.88	21.77±6.31	26.21±10.84	22.64±6.38	0.02	0.74	<0.01	0.02	0.01
ALT, U/L	50.42±17.03	28.74±15.04	26.81±10.63	24.46±12.01	36.35±16.28	25.91±13.22	0.01	0.21	<0.01	0.06	<0.01

a Comparison of the gender with NAFLD.

b Comparison of the gender with Non-NAFLD.

BMI, body mass index; BAZ, BMI-for-age; AST, aspartate aminotransferase; ALT, alanine aminotransferase; WC, waist circumference.

The NAFLD patients had significantly higher values for body weight, BAZ-score, BMI, total fat (% and kg), WC, visceral fat, insulin, HOMA-IR, AST and ALT, when compared with non-NAFLD obese adolescents. It is important to note that the mean±standard error of WC remained higher in the NAFLD *vs* non-NAFLD group (107.00±0.83 and 98.85±0.83; *p*<0.001, respectively), even after adjustments for possible confounders. In boys with NAFLD, body weight, BAZ-score, BMI, total fat (% and kg), WC, visceral fat, insulin, HOMA-IR, AST and ALT were significantly higher than the values found for non-NAFLD obese boys. In girls with NAFLD, values of body weight, BAZ-score, BMI, total fat (% and kg), WC, visceral fat, insulin and HOMA-IR were higher than those obtained in non-NAFLD obese girls (Table 1).

In obese boys with NAFLD, the values of body weight, fat free mass (% and kg), WC, visceral fat, HOMA-IR, ASL, and ALT are significantly higher when compared with girls of the same group. However, obese girls of NAFLD group had significantly more of total fat (47.71±4.84 *vs* 42.92±6.08%) than obese boys with NAFLD. Obese girls of group non-NAFLD presented lower values of body weight, fat free mass (% and kg), WC and AST when compared with obese non-NAFLD boys, but obese non-NAFLD girls had higher value of total fat (%) than obese non-NAFLD boys (Table 2).

**Table 2:** Anthropometric, subcutaneous and visceral adipose tissues, HOMA-IR and liver enzymes in obese adolescents according quartiles of waist circumference expressed as mean±standard deviation.

	Low: 1st (<91.8cm)			Moderate: 2nd (≥91.8–99cm)			High: 3rd (>99–107.5cm)			Very High: 4th (>107.5cm)	
	Boys (n=51)	Girls (n=11)		Boys (n=45)	Girls (n=17)		Boys (n=32)	Girls (n=30)		Boys (n=28)	Girls (n=33)
BAZ (Z score)	2.49±0.31	2.66±0.29		2.86±0.46	2.73±0.28		3.29±0.58	3.09±0.54		3.77±0.61	3.82±0.61
BMI (kg/m^2^)	31.49±2.41	30.54±2.71		35.01±3.24	32.67±1.82 a		38.09±4.06	35.32±3.66 a		41.46±4.19	40.15±4.11
Total fat (%)	39.10±6.07	42.17±4.53		46.44±4.79	37.84±5.89 a		48.70±4.39	39.16±6.15 a		49.76±4.17	43.97±5.94 a
WC (cm)	86.54±3.88	87.42±3.77		95.81±2.30	95.50±2.53		103.65±2.54	102.67±1.88		113.60±6.23	115.57±6.59
Visceral fat (cm)	3.48±1.12	3.96±1.17		4.06±1.06	3.76±1.15		4.19±1.52	5.07±1.33 a		4.69±1.20	5.75±1.71
Subcutaneous fat (cm)	3.17±0.82	2.42±0.66 a		3.66±0.72	3.44±0.83		4.37±1.03	3.58±0.68 a		4.38±1.22	3.85±1.02
HOMA-IR	3.82±1.75	3.31±1.95		3.49±1.44	4.02±2.78		4.01±1.68	4.16±1.69		4.75±1.88	5.31±3.14
AST U/L	22.88±7.54	20.81±3.02		21.06±4.42	24.76±7.07		21.43±4.74	30.80±13.90 a		21.39±4.42	29.48±10.43 a
ALT U/L	25.50±14.30	19.27±6.72		24.08±10.08	29.58±13.22		26.12±10.42	30.80±13.90 a		25.32±8.60	45.30±12.89 a

a Difference of the genders at the same quartile.

AST, aspartate aminotransferase; ALT, alanine aminotransferase; WC, waist circumference.

Differences between genders were found according to quartiles of WC (Table 2). In the first quartile, obese boys presented significantly higher values of subcutaneous fat (3.17±0.82 *vs* 2.42±0.66) when compared with obese girls. In the second quartile, higher values of BMI (35.01±3.24 *vs* 32.67±1.82) and total fat (46.44±4.79% *vs* 37.84±5.89%) were noted among obese boys than among obese girls. BMI, total fat and subcutaneous fat were also significantly higher in obese boys when compared with girls in the 3rd quartile (38.09±4.06kg/m^2^ *vs* 35.32kg/m^2^±3.66, 48.70±4.39% *vs* 39.16±6.15% and 4.37±1.03cm *vs* 3.58±0.68cm, respectively). Moreover, in the same quartile, higher values of visceral fat (4.19±1.52 *vs* 5.07±1.33cm), AST (21.43±4.74 *vs* 30.80±13.90U/L) and ALT (26.12±10.42 *vs* 30.80±13.90U/L) were observed in obese girls when compared with obese boys. Finally, in the 4th quartile in obese boys presented significantly higher values in total fat (49.76±4.17% *vs* 43.97±5.94%) and obese girls had significantly higher values of AST (21.39±4.42 *vs* 29.48±10.43U/L) and ALT (25.32±8.60 *vs* 45.30±26.89U/L).

Correlations between WC, biochemical and anthropometric parameters according to gender are shown in Table 3. In obese girls, the WC exhibited positive correlations with BAZ (*r* =0.72, *p*<0.01), BMI (*r* =0.76, *p* =0.01), total fat (%) (*r* =0.38, *p* =0.01), visceral fat (*r* =0.50; *p* =0.01), subcutaneous fat (*r* =0.42; *p* =0.01), HOMA-IR (*r* =0.38; *p* =0.01), AST (*r* =0.26; *p* =0.01) and ALT (*r* =0.32; *p* =0.02). In obese boys, WC positively correlated with BAZ (*r* =0.73; *p* =0.01), BMI (*r* =0.76; *p* =0.01), total fat (*r* =0.58; *p* =0.01), visceral fat (*r* =0.30; *p* =0.01) and subcutaneous fat (*r* =0.47; *p* =0.01).

**Table 3:** Correlations between waist circumference, biochemical and anthropometric parameters.

	Girls (n=157)			Boys (n=90)	
	Pearson R	*p* -value		Pearson R	*p* -value
BAZ (Z score)	0.722	0.001		0.731	0.001
BMI (kg/m^2^)	0.759	0.001		0.760	0.001
Total fat (%)	0.382	0.001		0.581	0.001
Visceral fat (cm)	0.504	0.001		0.306	0.001
Subcutaneous fat (cm)	0.424	0.001		0.470	0.001
HOMA-IR	0.384	0.001		0.128	0.111
AST, U/L	0.259	0.013		−0.041	0.610
ALT, U/L	0.324	0.002		0.109	0.615

AST, aspartate aminotransferase; ALT, alanine aminotransferase.

**Figure 2:**
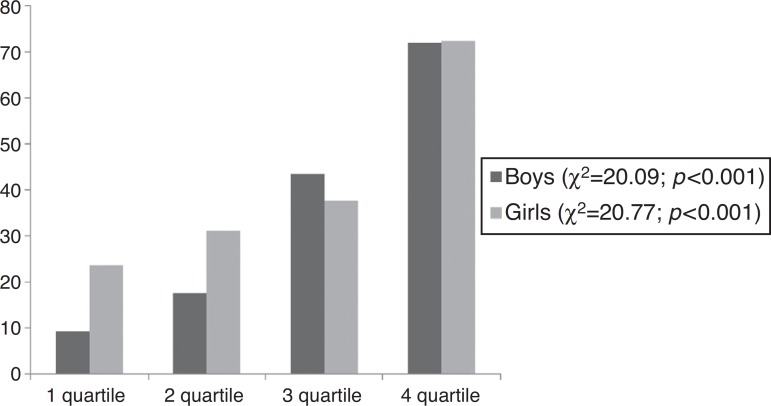
Prevalence of NAFLD according to quartiles of waist circumference.

Fig. 2 shows that the prevalence of NAFLD increases with the increase in the quartile of WC.

## Discussion

Therefore, the most important finding of the study was that 72.4% and 71.9% of girls and boys with NAFLD, respectively, were classified in the highest quartile of WC (WC>107.5cm). In agreement, the NAFLD adolescents had significantly higher values for body weight, BAZ-score, BMI, total fat (% and kg), WC, visceral fat, insulin, HOMA-IR, AST and ALT, when compared with non-NAFLD obese adolescents.

Corroborating with these results, it has been previously shown that both insulin resistance and abdominal adipose tissue are key risk factors to the development of NAFLD. Hepatic cirrhosis may be a consequence of the NAFLD in long-term and this may result in a higher risk of liver disease-related mortality, reinforcing the importance of the present investigation.^4^^,^ 8 ^,^ 17 ^,^ 18

Moreover, it is well known that individuals with increased abdominal fat are more susceptible to metabolic disorders, which are developed during childhood.^19^^,^ 20 In fact, in this study insulin resistance was higher in the 3rd and 4th quartiles when compared with the 1st quartile of WC in obese adolescents.

Furthermore, studies in adults have found that high levels of hepatic enzymes, particularly ALT, could predict insulin resistance and later development of type 2 diabetes mellitus. Other studies highlight that ALT seems to be an important marker of fatty liver disease in pediatric population.^21^^-^ 23 In our study, girls had significantly higher values of ALT in the 3rd and 4th quartile of WC when compared with boys. In the literature, another study showed the opposite: obese boys with NAFLD had higher ALT values than obese girls.^24^ Therefore, large cohort studies are needed to define gender differences in ALT in obese adolescents. Despite the controversy, the results suggest that sex hormones have a role in the manifestation of insulin resistance, beyond distribution of fat, muscle and binding globulin produced in the liver, which are strongly correlated with insulin sensitivity and the differences between ALT serum concentrations by gender.^24^

Importantly, it has been showed that abdominal obesity and NAFLD are strongly associated with cardiovascular disease.^9^^,^ 13 ^,^ 25 Also, previous research showed that visceral adiposity was closely related to NAFLD.^8^ In this way, both BMI and WC have been considered as predictors of NAFLD severity and independent predictors of steatosis.^25^Although biopsy is the gold standard technique for the diagnosis of NAFLD, this method is invasive and difficult to apply in clinical practice, especially in the pediatric population.^26^ Additionally, ultrasound imaging and magnetic resonance for diagnosis of NAFLD have some limitations, such as the high cost to be used as a screening method in developing countries. Thus, it is imperative to develop simple and sensitivity indicators to identify NAFLD.^13^^,^ 14

Recently, WC has been considered as a potential screening tool for liver steatosis and cardiovascular risk, being the most cost-effective and feasible replacement for ultrasound and magnetic resonance in the assessment of NAFLD in obese adolescents.^27^ Our results reinforce this finding, suggesting that WC >99cm (3rd quartile) as a cutoff for detection of NAFLD and metabolic alterations. In addition, we demonstrated that the obese adolescents in the 3rd and 4th quartiles of WC presented higher prevalence of NAFLD when compared with those on the 1st quartile, for both genders. These results show the importance of this anthropometric measurement to detect incremental risk of steatosis in the analyzed population.

Together these results reinforce that WC is simple to measure and can be applied as an important anthropometric indicator of central obesity to screen adolescents with high risk for NAFLD. The measure of WC is considered a new risk factor for metabolic syndrome, with advantages for the diagnosis and follow-up of the treatment in NAFLD patients.^28^ Although, more researches with different populations are needed to confirm the external validity of the obtained results, this study suggests that the use of such a marker in clinical practice could be valuable since anthropometrical measurements are inexpensive and straightforward.^11^

The criteria for diagnosis of metabolic syndrome in adolescents adopted from the International Diabetes Federation (IDF) considers WC greater than 80cm for girls and 94cm for boys have high sensitivity for screening adolescents at risk of metabolic disorders.^12^ Another study with adults showed that appropriate cutoff points of WC for detecting NAFLD were 89cm for men and 84cm for women with high negative predictive values for NAFLD.^29^ These results confirm the importance of using WC in clinical practice, as it may contribute to evaluate the risk of NAFLD and insulin resistance. In this way, the identification of threshold values for WC in children and adolescents is a crucial component in developing a strategy for the prevention of metabolic diseases as NAFLD in overweight subjects. Therefore, the major finding of this study is that WC is a convenient measure of abdominal obesity associated with the risk of NAFLD development in obese adolescents. This tool requires only the purchase of an appropriate tape measure and simple training of health professionals and/or assistants. It can be easily incorporated in the assessment of children and adolescents at the time the body weight is obtained. The implementation of preventive measures among vulnerable populations would ensure a better quality of life and would serve to minimize future spending by health care systems.

There are some limitations to this study. Because of its cross sectional design, our study does not provide evidence of a cause and effect relationship. WC values proposed in this study cannot be generalized to other populations, more researches with different populations are needed to confirm the external validity of the obtained results. Despite these limitations, our research indicates that higher WC may be a significant risk factor for the development of metabolic disorders. In particular, the values of WC found in the 3rd and 4th quartiles for girls and boys might be a reliable screening tool for NAFLD risk in obese adolescents. The present findings suggest the need for further longitudinal studies, with larger samples, from different geographical/socio-cultural environments. In conclusion, the results presented here suggest that an increase in WC can reliably predict the risk of NAFLD in obese adolescents. This is a low cost and easy-to-use tool that can help in screening metabolic risk factors in adolescents.
