# Supplementary material for: Waist circumference as a marker for screening nonalcoholic fatty liver disease in obese adolescents
Source: Rev Paul Pediatr. 2016 Jan-Mar;34(1):47–55. doi: 10.1016/j.rppede.2015.10.004 (PMC4795721; doi:10.1016/j.rppede.2015.10.004)
Supplement: Translation

# Translation: Circunferência da cintura como marcador para triagem de doença hepática gordurosa não alcoólica em adolescentes obesos

**Authors:** Ana Paula Grotti Clemente, Bárbara Dal Molin, Joana Pereira de Carvalho-Ferreira, Raquel Munhoz da Silveira Campos, Aline de Piano Ganen, Lian Tock, Marco Túlio de Mello, Ana Raimunda Dâmaso

**Affiliations:** a Universidade Federal de Alagoas (Ufal), Maceió, AL, Brasil; b Escola Paulista de Medicina, Universidade Federal de São Paulo (EPM-Unifesp), São Paulo, SP, Brasil

**Keywords:** Aspartato aminotransferase, Alanina aminotransferase, Gordura abdominal, Adolescentes

## Abstract

**Objetivo:**

Avaliar a associação entre o grau de circunferência da cintura (CC) e doença hepática gordurosa não alcoólica (DHGNA) em adolescentes obesos de ambos os sexos, avaliados de acordo com quartis de CC.

**Métodos:**

Estudo transversal que envolveu 247 adolescentes obesos entre 12 a 19 anos. Os valores médios dos parâmetros nutricionais e as analises séricas foram comparados com o teste *t* independente. O coeficiente de correlação de Pearson foi usado para determinar a relação entre os parâmetros estudados. O teste do qui-quadrado de tendência foi usado para determinar a relação entre a prevalência da DHGNA e quartil da CC por sexo.

**Resultados:**

DHGNA estava presente em 60% dos participantes do estudo. Adolescentes obesos nos quartis 3 e 4 de CC apresentaram maior prevalência de DHGNA quando comparados com aqueles no primeiro quartil em ambos os sexos. Os pacientes com DHGNA tinham valores significativamente mais elevados de peso corporal, IMC (índice de massa corporal), IMC/I (IMC para idade) z-escore, gordura total (% e kg), CC, gordura visceral, insulina, índice de resistência à insulina (HOMA-IR), aspartato aminotransferase e alanina aminotransferase quando comparados com adolescentes obesos sem DHGNA.

**Conclusões:**

Os resultados aqui apresentados sugerem que um aumento da CC pode predizer com segurança o risco de DHGNA em adolescentes obesos. Essa é uma ferramenta de baixo custo e fácil de usar que pode ajudar na triagem de adolescentes.

## Introdução

A doença hepática gordurosa não alcoólica (DHGNA) é a causa mais comum de doença crônica hepática em todo o mundo e tem sido reconhecida como manifestação precoce de obesidade e síndrome metabólica.^1^ A DHGNA caracteriza-se pela acúmulo de grandes gotículas de triglicérides no interior dos hepatócitos na ausência de consumo crónico de álcool.^2^ Atualmente, a DHGNA afeta entre 3% e 11% da população pediátrica, alcança a taxa de 46% entre as crianças e adolescentes com sobrepeso e obesidade.^3^ De fato, um estudo prévio de nosso grupo descobriu que a DHGNA afetava 52% dos adolescentes obesos.^4^

O desenvolvimento da DHGNA é influenciado por múltiplos fatores genéticos e ambientais. Atualmente, a DHGNA é reconhecida como o componente hepático da síndrome metabólica, devido à sua forte associação com a obesidade, dislipidemia, hipertensão arterial e índice de resistência à insulina (HOMA-IR). Sabe-se há muito tempo que há uma relação altamente significativa entre DHGNA e a resistência à insulina. Um estudo desenvolvido com crianças japonesas sugeriu que a hiperinsulinemia era a manifestação clínica mais importante associada com a DHGNA.^5^ Além disso, a resistência à insulina é aceita como o principal fator fisiopatológico no desenvolvimento da DHGNA.^6^

Da mesma forma, Piano et al.^7^ verificaram que adolescentes com obesidade visceral e níveis elevados de HOMA-IR apresentavam maior risco de desenvolver a DHGNA, o que poderia levar ao acúmulo de lípides nos hepatócitos. Além disso, demonstrou-se que cada aumento de 1cm na adiposidade visceral estava associado com um risco duas vezes maior de DHGNA em adolescentes obesos.^8^

De fato, a adiposidade central está associada com inflamação crônica de baixo grau, o que acelera a resistência à insulina e o acúmulo de gordura hepatocelular. Indivíduos com DHGNA estão em risco de desenvolver doenças cardiovasculares (DCV) por meio de mecanismos relacionados com a resistência à insulina.^9^ Portanto, é importante avaliar a adiposidade visceral na prática clínica. Para a avaliação da obesidade central em idades mais jovens, a ultrassonografia e a ressonância magnética estão disponíveis. No entanto, esses procedimentos têm algumas limitações para o seu uso amplo, como, por exemplo, o custo. Por outro lado, a CC pode ser uma ferramenta clínica simples e de baixo custo para ser usada como um marcador substituto para a DHGNA.^10^ A CC tem demonstrado ser uma ferramenta de baixo custo para avaliar a obesidade central na prática clínica, com excelente correlação com exames de imagem abdominais e alta associação com risco de DCV.^11^ Por essa razão, a CC é um dos critérios de diagnóstico propostos pela International Diabetes Federation (IDF) em adolescentes e tem sido identificada como um preditor importante da síndrome metabólica e risco de DCV.^12^

A relação entre DHGNA e o desenvolvimento da aterosclerose foi avaliada em estudos pediátricos.^1^^,^ 9 Fallo et al.^13^ relataram que a CC foi um preditor de DHGNA em seu estudo que incluiu 86 adultos obesos hipertensos. Outro estudo encontrou que o aumento da CC e do índice de massa corporal (IMC) estavam associadas com um risco significativamente maior de resistência à insulina e DHGNA em adultos coreanos saudáveis. Além disso, os autores reforçaram a importância de usar tanto o IMC quanto a CC na prática clínica, porque eles podem ser úteis para avaliar o risco de DHGNA e de resistência à insulina.^14^ Finalmente, sabe-se que a medida da CC prevê o risco cardiovascular, embora o seu valor para a avaliação de risco da DHGNA em adolescentes ainda não tenha sido explorado.

Portanto, a CC é uma medida conveniente de obesidade abdominal. Entretanto, poucos estudos têm sido feitos sobre a relação entre a área de gordura intra-abdominal e risco de DHGNA. Dessa forma, nosso estudo teve como objetivo avaliar a relação entre a CC e a presença de DHGNA em adolescentes obesos brasileiros de ambos os sexos, analisados de acordo com quartis de CC.

## Método

O estudo foi aprovado pelo Comitê de Ética em Pesquisa da Universidade Federal de São Paulo (Unifesp; Protocolo n° 0135/04) e registrado como um estudo clínico (NCT01358773). O consentimento informado foi obtido de todos os potenciais participantes e/ou seus pais ou responsáveis legais antes do início do estudo.

O fluxograma do estudo é mostrado na fig. 1. Para este estudo transversal, foram incluídos 247 adolescentes obesos entre 12 e 19 anos. Os dados foram coletados por meio da triagem de adolescentes obesos em 2007-2010. Os adolescentes obesos foram recrutados no Programa Ambulatorial de Intervenção Multidisciplinar em Obesidade da Universidade Federal de São Paulo. Todos os pacientes incluídos neste estudo foram avaliados antes da terapia de perda de peso. O estado nutricional foi calculado de acordo com valores de escore Z da estatura para a idade (HAZ) e IMC para idade (BAZ) com o *software* WHO Anthro-Plus 1.0.4. O diagnóstico nutricional foi baseado no IMC-para-idade (BAZ) para as crianças com idade superior a cinco anos e adolescentes com ≤19 anos (escore Z +2DP), de acordo com os pontos de corte definidos pela Organização Mundial da Saúde.^16^ Os critérios de não inclusão foram identificados como doenças genéticas, metabólicas ou endócrinas, consumo crônico de álcool (≥20g/dia), presença de doenças hepáticas virais, uso anterior de drogas e outras causas de esteatose hepática.

**Figura 1:** Descrição do fluxograma do estudo. ECG, eletrocardiograma; GEO, Grupo de Estudo em Obesidade; AST, aspartato aminotransferase; ALT, alanina aminotransferase.

O desenvolvimento puberal foi estabelecido com a triagem clínica e foram avaliadas as medidas antropométricas (estatura, massa corporal, IMC e composição corporal). A ultrassonografia (US) foi feita e a amostra de sangue foi colhida e analisada para o perfil metabólico. Para excluir influências de variações diurnas, os procedimentos foram programados para o mesmo horário do dia para todos os indivíduos, às 8h, após um jejum noturno.

Todos os adolescentes foram examinados por um médico treinado e o estágio puberal foi classificado de acordo com a escala de Tanner^15^ para meninos e meninas. Meninas com mamas em estágio 2 e meninos com genitália em estágio 3 foram considerados puberais, enquanto aqueles que ainda não tinham atingido esses estágios foram classificados como não puberais.^15^ Nenhum dos participantes apresentava puberdade precoce ou atrasada, embora os níveis de testosterona, hormônio luteinizante ou hormônio folículo-estimulante não tenham sido determinados.

A massa corporal foi medida (com roupas leves e sem sapatos) em uma única avaliação, com uma balança de plataforma da marca Filizola^®^ (Indústrias Filizola S/A, São Paulo-SP, Brasil; modelo PL 180), com capacidade de 180kg e precisão de 100g. A estatura foi avaliada com um estadiômetro com precisão de 0,1cm (Sanny, São Bernardo do Campo, SP, Brasil; modelo ES 2030). Os valores de IMC foram calculados pelo quociente da massa corporal (kg) e o quadrado da estatura (m). Para a determinação da CC, os indivíduos foram colocados em posição ereta com o abdômen e os braços relaxados ao longo do corpo e uma fita métrica flexível (precisão 1mm) foi colocada horizontalmente no ponto médio entre a borda inferior da última costela e a crista ilíaca. As medidas foram feitas com a fita aplicada firmemente à pele, mas sem compressão de tecidos. A composição corporal foi medida por meio de pletismografia por deslocamento de ar em um sistema de composição corporal BOD POD (versão 1.69; Life Measurement Instruments, Concord, CA).

Amostras de sangue (10mL) foram coletadas dos adolescentes em jejum noturno por punção venosa e transferidas, conforme apropriado, para tubos de ensaio heparinizados e não heparinizados. A glucose no plasma foi determinada com o auxílio de um kit comercial e um espectrofotômetro Unicell DXI 800 (Beckman Coulter, Fullerton, CA, EUA), enquanto que a insulina específica (sem peptídeo C) foi determinada com um ensaio enzimático e um analisador Advia 2400/Kovalent (Siemens, São Paulo, Brasil). Os níveis séricos das transaminases hepáticas, da alanina aminotransferase (ALT) e dp aspartato aminotransferase (AST) foram analisados com um kit comercial (CELM, Barueri, Brasil). A resistência à insulina foi avaliada pelo modelo de avaliação da homeostase de resistência à insulina (HOMA-IR). O índice HOMA-IR foi calculado pela glicemia de jejum (GJ) no sangue e a insulina imunorreativa (I): [GJ (mg/dL) I (mU/L)]/405.

Adolescentes obesos foram divididos em quartis de acordo com a CC. No primeiro quartil ficaram os adolescentes com menos de 91,8cm de CC (CC baixa); no segundo quartil, adolescentes com valores de CC entre 91,8 e 99cm (CC moderada); no terceiro quartil, adolescentes com CC entre 99,1 e 107,5cm (WC alta); finalmente, no quarto quartil, foram incluídos adolescentes com CC acima de 107,5cm.

As análises estatísticas foram feitas com o *software* PASW Statistics, versão 19 (SPSS Inc, Chicago, IL, EUA), com um nível de significância de *p*<0,05. Os valores médios dos parâmetros nutricionais (idade, altura, peso, HAZ, IMC e CC) e as análises séricas (insulina, glicemia, ALT e AST) dos grupos não DHGNA e DHGNA, estratificados de acordo com sexo, foram comparados com o teste *t* independente e o pressuposto da homocedasticidade verificado pelo teste de Levene. O coeficiente de correlação de Pearson foi usado para determinar a relação entre a variável independente estado nutricional e os parâmetros bioquímicos e CC. O teste qui-quadrado para tendência foi usado para determinar a relação entre a prevalência da DHGNA e o quartil da CC por sexo. Finalmente, foi feita uma análise de covariância com a presença de DHGNA como fator e CC como a variável dependente. Já que outras variáveis antropométricas apresentaram um alto grau de correlação com CC, os efeitos confundidores de idade e sexo foram avaliados no grupo com DHGNA.

## Resultados

O estudo incluiu 247 adolescentes obesos: 90 meninos (36,5%) e 157 meninas (63,5%). Entre os participantes, 148 (60%) apresentavam DHGNA. A composição corporal e as características antropométricas e bioquímicas dos indivíduos são apresentadas na tabela 1.

**Tabela 1:** Dados antropométricos, composição corporal e parâmetros bioquímicos da população estudada

	DHGNA (n=40)	Não DHGNA (n=50)	DHGNA (n=59)	Não DHGNA (n=98)	NAFLD (n=148)	Não NAFLD (n=99)	*p* ‐valor Meninos	*p* ‐valor Meninas	Grupo de Gênero a	Grupo de Gênero b	Total
Idade (anos)	16,78±1,63	15,87±1,65	16,94±2,10	16,30±1,80	16,88±1,92	16,15±1,76	0,01	0,04	0,67	0,15	0,02
Peso (kg)	117,07±17,24	100,48±16,08	100,69±15,45	91,13±13,75	107,31±18,02	94,29±15,19	<0,01	<0,01	<0,01	<0,01	<0,01
Estatura (cm)	174,65±6,77	171,34±8,18	163,37±6,85	162,53±5,93	167,92±8,77	165,50±7,93	0,04	0,41	<0,01	<0,01	0,03
BAZ (Z‐escore)	3,51±0,83	3,04±0,65	3,25±0,80	2,82±0,69	3,36±0,82	2,89±0,68	0,04	0,01	0,07	0,23	<0,01
IMC (kg/m^2^)	38,37±4,95	34,08±4,10	37,71±4,98	34,46±4,52	37,97±4,95	34,33±4,38	<0,01	<0,01	0,52	0,64	<0,01
Gordura Total (%)	42,92±6,08	38,08±6,04	47,71±4,84	45,20±5,54	45,77±5,84	42,97±6,49	0,01	0,05	<0,01	<0,01	0,01
Massa livre de gordura (%)	57,32±5,98	61,50±6,18	52,28±4,84	54,92±5,43	54,32±5,85	57,16±6,48	0,02	0,03	<0,01	<0,01	0,01
Gordura Total (kg)	50,72±12,25	38,96±9,96	48,35±10,47	41,37±10,28	49,31±11,22	40,55±10,20	<0,01	<0,01	0,31	0,17	<0,01
Massa livre de gordura (kg)	66,52±9,76	62,58±10,95	52,28±6,97	49,31±5,02	58,04±10,77	53,82±9,83	0,08	0,05	<0,01	<0,01	0,02
CC (cm)	110,30±10,83	99,34±8,17	102,59±10,82	94,71±9,05	105,70±11,42	96,27±9,01	<0,01	<0,01	0,01	0,03	<0,01
Gordura Visceral (cm)	5,82±1,64	4,30±1,23	4,52±1,31	3,73±1,20	5,05±1,58	3,92±1,24	<0,01	<0,01	<0,01	0,22	<0,01
Gordura Subcutânea (cm)	3,60±0,95	3,44±0,88	3,86±1,09	3,69±1,04	3,75±1,04	3,60±1,00	0,41	0,33	0,27	0,07	0,26
Glicose (mg/dL)	92,17±7,99	91,54±6,47	89,91±7,52	89,93±6,58	90,82±7,75	90,47±6,56	0,68	0,98	0,15	0,16	0,70
Insulina (uU/mL)	23,87±12,42	15,98±10,28	20,00±8,33	16,10±10,37	21,56±10,30	16,06±10,30	<0,01	0,01	0,06	0,95	<0,01
HOMA‐IR	5,47±2,91	3,64±2,51	4,37±1,90	3,66±2,94	4,81±2,41	3,65±2,79	0,02	0,01	0,04	0,98	0,01
AST, U/L	32,30±14,00	24,36±6,23	22,08±4,88	21,77±6,31	26,21±10,84	22,64±6,38	0,02	0,74	<0,01	0,02	0,01
ALT, U/L	50,42±17,03	28,74±15,04	26,81±10,63	24,46±12,01	36,35±16,28	25,91±13,22	0,01	0,21	<0,01	0,06	<0,01

a Comparação do sexo com DHGNA.

b Comparação do sexo com não DHGNA.

IMC, índice de massa corporal; BAZ, IMC para idade; AST, aspartato aminotransferase; ALT, alanina aminotransferase; CC, circunferência da cintura,

Os pacientes com DHGNA tinham valores significativamente mais elevados de peso corporal, BAZ, IMC, gordura total (% e kg), CC, gordura visceral, insulina, HOMA-IR, AST e ALT, quando comparados com os adolescentes obesos não DHGNA. É importante notar que a média ± erro padrão da CC permaneceram mais elevados no grupo DHGNA *vs*. não DHGNA (107±0,83 e 98,85±0,83; *p*<0,001, respectivamente), mesmo após o ajuste para possíveis variáveis confundidoras. Em meninos com DHGNA, peso corporal, BAZ, IMC, gordura total (% e kg), CC, gordura visceral, insulina, HOMA-IR, AST e ALT foram significativamente maiores do que os valores encontrados para os meninos obesos não DHGNA. Nas meninas com DHGNA, os valores de peso corporal, BAZ, IMC, gordura total (% e kg), CC, gordura visceral, insulina e HOMA-IR foram maiores do que os obtidos em meninas obesas não DHGNA (tabela 1).

Nos meninos obesos com DHGNA, os valores de peso corporal, massa livre de gordura (% e kg), CC, gordura visceral, HOMA-IR, ASL, e ALT são significativamente mais elevados quando comparados com os das meninas do mesmo grupo. No entanto, as meninas obesas do grupo DHGNA tinham significativamente mais gordura total (47,71±4,84 *vs*. 42,92±6,08%) do que os meninos obesos com DHGNA. Meninas obesas do grupo não DHGNA apresentavam valores menores de peso corporal, massa livre de gordura (% e kg), CC e AST quando comparadas com meninos obesos não DHGNA, mas meninas obesas não DHGNA tinham valor mais alto de gordura total (%) do que os meninos obesos não DHGNA (tabela 2).

**Tabela 2:** Dados antropométricos, tecido adiposo subcutâneo e visceral, HOMA‐IR e enzimas hepáticas em adolescentes obesos, segundo os quartis da circunferência da cintura expressos em média±desvio padrão

	Baixo: 1^o^ (<91,8cm)			Moderado: 2^o^ (≥91,8‐99cm)			Alto: 3^o^ (>99‐107,5cm)			Muito alto:4^o^ (>107,5cm)	
	Meninos (n=51)	Meninas (n=11)		Meninos (n=45)	Meninas (n=17)		Meninos (n=32)	Meninas (n=30)		Meninos (n=28)	Meninas (n=33)
BAZ (escore Z)	2,49±0,31	2,66±0,29		2,86±0,46	2,73±0,28		3,29±0,58	3,09±0,54		3,77±0,61	3,82±0,61
IMC (kg/m 2 )	31,49±2,41	30,54±2,71		35,01±3,24	32,67±1,82 a		38,09±4,06	35,32±3,66 a		41,46±4,19	40,15±4,11
Gordura total (%)	39,10±6,07	42,17±4,53		46,44±4,79	37,84±5,89 a		48,70±4,39	39,16±6,15 a		49,76±4,17	43,97±5,94 a
CC (cm)	86,54±3,88	87,42±3,77		95,81±2,30	95,50±2,53		103,65±2,54	102,67±1,88		113,60±6,23	115,57±6,59
Gordura visceral (cm)	3,48±1,12	3,96±1,17		4,06±1,06	3,76±1,15		4,19±1,52	5,07±1,33 a		4,69±1,20	5,75±1,71
Gordura subcutânea (cm)	3,17±0,82	2,42±0,66 a		3,66±0,72	3,44±0,83		4,37±1,03	3,58±0,68 a		4,38±1,22	3,85±1,02
HOMA‐IR	3,82±1,75	3,31±1,95		3,49±1,44	4,02±2,78		4,01±1,68	4,16±1,69		4,75±1,88	5,31±3,14
AST U/L	22,88±7,54	20,81±3,02		21,06±4,42	24,76±7,07		21,43±4,74	30,80±13,90 a		21,39±4,42	29,48±10,43 a
ALT U/L	25,50±14,30	19,27±6,72		24,08±10,08	29,58±13,22		26,12±10,42	30,80±13,90 a		25,32±8,60	45,30±12,89 a

a Diferença entre os sexos no mesmo quartil.

AST, aspartato aminotransferase; ALT, alanina aminotransferase; CC, circunferência da cintura.

As diferenças entre os sexos foram encontradas de acordo com os quartis de CC (tabela 2). No primeiro quartil, meninos obesos apresentaram valores significativamente mais elevados de gordura subcutânea (3,17±0,82 *vs*. 2,42±0,66) em comparação com meninas obesas. No segundo quartil, valores mais altos de IMC (35,01±3,24 *vs*. 32,67±1,82) e de gordura total (46,44±4,79% *vs*. 37,84±5,89%) foram observados nos meninos obesos do que nas meninas obesas. IMC, gordura total e gordura subcutânea também foram significativamente maiores nos meninos obesos quando comparados com as meninas no terceiro quartil (38,09±4,06kg/m^2^ *vs*. 35,32±3,66kg/m^2^, 48,70±4,39% *vs*. 39,16±6,15% e 4,37±1,03cm *vs*. 3,58±0,68cm, respectivamente). Além disso, no mesmo quartil, valores mais elevados de gordura visceral (4,19±1,52 *vs*. 5,07±1,33cm), AST (21,43±4,74 *vs.* 30,80±13,90U/L) e ALT (26,12±10,42 *vs.* 30,80±13,90U/L) foram observados em meninas obesas quando comparadas com os meninos obesos. Finalmente, no quarto quartil, meninos obesos apresentaram valores significativamente mais elevados de gordura total (49,76±4,17% *vs.* 43,97±5,94%) e meninas obesas apresentaram valores significativamente mais altos de AST (21,39±4,42 *vs.* 29,48±10,43U/L) e ALT (25,32±8,60 *vs.* 45,30±26,89U/L).

As correlações entre CC, parâmetros bioquímicos e antropométricos de acordo com o sexo são apresentadas na tabela 3. Nas meninas obesas, a CC apresentou correlações positivas com BAZ (r=0,72, *p*<0,01), IMC (r=0,76, *p*=0,01), gordura total (%) (r=0,38, *p*=0,01), gordura visceral (r=0,50; *p*=0,01), gordura subcutânea (r=0,42; *p*=0,01), HOMA-IR (r=0,38; *p*=0,01), AST (r=0,26; *p*=0,01) e ALT (r=0,32; *p*=0,02). Em meninos obesos, a CC foi positivamente correlacionada com BAZ (r=0,73; *p*=0,01), IMC (r=0,76; *p*=0,01), a gordura total (r=0,58; *p*=0,01), a gordura visceral (r=0,30; *p*=0,01) e gordura subcutânea (r=0,47; *p*=0,01).

**Tabela 3:** Correlações entre a circunferência da cintura, parâmetros antropométricos e bioquímicos

	Meninas (n=157)			Meninos (n=90)	
	Pearson R	*p* ‐valor		Pearson R	*p* ‐valor
BAZ (escore Z)	0,722	0,001		0,731	0,001
IMC (kg/m^2^)	0,759	0,001		0,760	0,001
Gordura Total (%)	0,382	0,001		0,581	0,001
Gordura Visceral (cm)	0,504	0,001		0,306	0,001
Gordura Subcutânea (cm)	0,424	0,001		0,470	0,001
HOMA‐IR	0,384	0,001		0,128	0,111
AST, U/L	0,259	0,013		−0,041	0,610
ALT, U/L	0,324	0,002		0,109	0,615

AST, aspartato aminotransferase; ALT, alanina aminotransferase.

A figura 2 mostra que a prevalência da DHGNA aumenta com o aumento no quartil de CC.

**Figura 2:** Prevalência de DHGNA de acordo com os quartis de circunferência da cintura.

## Discussão

O achado mais importante do estudo foi que 72,4% e 71,9% das meninas e dos meninos com DHGNA, respectivamente, foram classificados no quartil mais alto de CC (CC>107,5cm). Em conformidade, os adolescentes com DHGNA tiveram valores significativamente mais elevados de peso corporal, BAZ, IMC, gordura total (% e kg), CC, gordura visceral, insulina, HOMA-IR, AST e ALT quando comparados com os adolescentes obesos não DHGNA.

Corroborando esses resultados, foi anteriormente demonstrado que tanto a resistência à insulina quanto o tecido adiposo abdominal são fatores de risco importantes para o desenvolvimento de DHGNA. A cirrose hepática pode ser uma consequência da DHGNA em longo prazo e isso pode resultar em um maior risco de mortalidade por doença hepática e reforçar a importância do presente estudo.^4^^,^ 8 ^,^ 17 ^,^ 18

Além disso, é sabido que os indivíduos com aumento da gordura abdominal são mais suscetíveis a doenças metabólicas, que se desenvolvem durante a infância.^19^^,^ 20 De fato, neste estudo a resistência à insulina foi maior no 3° e 4° quartis, quando comparada com o primeiro quartil de CC em adolescentes obesos.

Do mesmo modo, estudos em adultos descobriram que os níveis elevados de enzimas hepáticas, particularmente ALT, poderiam prever a resistência à insulina e posterior desenvolvimento da *diabetes mellitus* tipo 2. Outros estudos destacam que a ALT parece ser um importante marcador de doença hepática gordurosa na população pediátrica.^21^^-^ 23 Em nosso estudo, as meninas tinham valores significativamente mais altos de ALT nos quartis 3 e 4 da CC quando comparadas com os meninos. Na literatura, outro estudo mostrou o contrário: os meninos obesos com DHGNA apresentavam valores de ALT mais elevados do que as meninas obesas.^24^ Portanto, grandes estudos de coorte são necessários para definir as diferenças de gênero em relação à ALT em adolescentes obesos. Apesar da controvérsia, os resultados sugerem que os hormônios sexuais têm um papel na manifestação da resistência à insulina além da distribuição de gordura, músculo e globulina de ligação produzida no fígado, que estão fortemente correlacionados com a sensibilidade à insulina e as diferenças entre as concentrações séricas de ALT por sexo.^24^

É importante lembrar que foi demonstrado que a obesidade abdominal e DHGNA estão fortemente associadas com doença cardiovascular.^9^^,^ 13 ^,^ 25 Além disso, pesquisas anteriores mostraram que a adiposidade visceral foi estreitamente relacionada com DHGNA.^8^ Dessa forma, tanto o IMC quanto a CC foram considerados como preditores de gravidade da DHGNA e preditores independentes de esteatose.^25^ Embora a biópsia seja a técnica padrão-ouro para o diagnóstico da DHGNA, esse método é invasivo e de difícil aplicação na prática clínica, especialmente na população pediátrica.^26^ Além disso, os exames de imagem por ultrassom e ressonância magnética para diagnóstico de DHGNA têm algumas limitações, tais como o elevado custo para ser usados como um método de triagem nos países em desenvolvimento. Assim, é imperativo desenvolver indicadores simples e com sensibilidade para identificar a DHGNA.^13^^,^ 14

Recentemente, a CC tem sido considerada como uma ferramenta de triagem potencial para a esteatose hepática e risco cardiovascular, é um substituto mais custo-efetivo e viável em comparação com a ultrassonografia e a ressonância magnética na avaliação da DHGNA em adolescentes obesos.^27^ Nossos resultados reforçam esse achado, sugerem a CC >99 cm (3° quartil) como ponto de corte para a detecção de DHGNA e alterações metabólicas. Além disso, foi demonstrado que os adolescentes obesos nos quartis 3 e 4 da CC apresentavam maior prevalência de DHGNA quando comparados com aqueles no primeiro quartil, para ambos os sexos. Esses resultados mostram a importância dessa medida antropométrica para detectar aumento do risco de esteatose na população analisada.

Juntos, esses resultados reforçam que a CC é simples de medir e pode ser aplicada como um importante indicador antropométrico de obesidade central para a triagem de adolescentes com alto risco de DHGNA. A medida de CC é considerada um novo fator de risco para a síndrome metabólica, com vantagens para o diagnóstico e acompanhamento do tratamento em pacientes com DHGNA.^28^ Embora, mais pesquisas com diferentes populações sejam necessárias para confirmar a validade externa dos resultados obtidos, este estudo sugere que o uso de tal marcador na prática clínica pode ser valioso, já que as medidas antropométricas são de baixo custo e simples de usar.^11^

Os critérios adotados para diagnóstico de síndrome metabólica em adolescentes, de acordo com a International Diabetes Federation (IDF) considera a CC maior do que 80 cm para as meninas e 94 cm para os meninos como tendo alta sensibilidade para a identificação de adolescentes em risco de distúrbios metabólicos.^12^ Outro estudo com adultos mostrou que os pontos de corte adequados da CC para a detecção de DHGNA foram 89 cm para homens e 84 cm para mulheres, com valores preditivos negativos elevados para DHGNA.^29^ Esses resultados confirmam a importância do uso de CC na prática clínica, uma vez que podem contribuir para avaliar o risco de esteatose hepática e resistência à insulina. Desse modo, a identificação dos valores de limiar para CC em crianças e adolescentes é um componente crucial no desenvolvimento de uma estratégia para a prevenção de doenças metabólicas como DHGNA em indivíduos com sobrepeso. Portanto, a principal conclusão desse estudo é que a CC é uma medida conveniente da obesidade abdominal associada com o risco de desenvolvimento de esteatose hepática em adolescentes obesos. Essa ferramenta requer apenas a compra de uma fita métrica adequada e o treinamento simples de profissionais e/ou assistentes de saúde. Ela pode ser facilmente incorporada na avaliação de crianças e adolescentes no momento em que o peso corporal é obtido. A implantação de medidas preventivas em populações vulneráveis garantiria uma melhor qualidade de vida e serviria para minimizar gastos futuros pelos sistemas de cuidados de saúde.

Este estudo tem algumas limitações. Devido ao seu desenho transversal, não fornece evidência de uma relação de causa e efeito. Os valores de CC propostos neste estudo não podem ser generalizados para outras populações e mais estudos com diferentes populações são necessários para confirmar a validade externa dos resultados obtidos. Apesar dessas limitações, nossa pesquisa indica que uma CC elevada pode ser um fator de risco significativo para o desenvolvimento de distúrbios metabólicos. Em particular, os valores de CC encontrados nos 3° e 4° quartis para meninas e meninos pode ser uma ferramenta de triagem confiável para o risco de DHGNA em adolescentes obesos. Os achados sugerem a necessidade de mais estudos longitudinais, com amostras maiores, de diferentes ambientes geográficos/socioculturais. Em conclusão, os resultados aqui apresentados sugerem que um aumento da CC pode prever com segurança o risco de DHGNA em adolescentes obesos. Essa é uma ferramenta de baixo custo e fácil de usar que pode ajudar na triagem de fatores de risco metabólicos em adolescentes.
